# The adherence to guidelines for preventing CVC-related infections: a survey among Italian health-care workers

**DOI:** 10.1186/s12879-018-3514-x

**Published:** 2018-12-03

**Authors:** Pietro Ferrara, Luciana Albano

**Affiliations:** Department of Experimental Medicine, University of Campania “Luigi Vanvitelli”, 5, Via Luciano Armanni, 80138 Naples, Italy

**Keywords:** Central venous catheters, CLABSIs, Evidence based practices, Guidelines adherence

## Abstract

**Background:**

Since correct maintenance of intravascular catheters is an effective strategy for preventing central-line infections, the aim of this study was to assess the level of adherence to guidelines for CVC maintenance amongst Italian HCWs.

**Methods:**

From July 2016 thru January 2017, a cross sectional survey was carried out in a random sample of 549 HCWs working in different hospitals of Campania region (Italy).

**Results:**

The 68.9% of interviewees returned the questionnaire. Overall, respondents’ level of knowledge about CDC guidelines was low, with only the 20.7% of HCWs acknowledging guidelines main recommendations: the nurse stuff, the availability of hospital internal protocols, the use of guidelines themselves as source of information, and higher number of years of practice were significantly associated with a higher level of knowledge. An extremely positive attitude towards the utility of guidelines for preventing CVC-related infections was shown, with a linear regression model indicating a stronger attitude in physicians, in who knew the CDC main recommendations and correct use of antibiotic ointments, as well as in HCWs needing additional information on the prevention of CVC-related infections. Regarding the behaviors, physicians were more likely to be adherent about recommended evidence-based practices. Two more multivariate logistic and ordinal logistic regression models were built to investigate characteristics associated with correct behavior regarding the removal of catheter dressing if patients have tenderness at insertion site or fever without an obvious source, respectively.

**Conclusions:**

This study reflected an important lack of evidence-based knowledge and practices regarding the CVC management, highlighting the baseline role of education and training programs, as well as pointing out the role of organizational interventions to address the adherence to best practices for the reduction of CLABSIs.

**Electronic supplementary material:**

The online version of this article (10.1186/s12879-018-3514-x) contains supplementary material, which is available to authorized users.

## Background

Central venous catheters (CVCs) are used in the patient care for the administration of intravenous fluids, medications, blood products, and parenteral nutrition, as well as for provide access for hemodialysis and hemodynamic monitoring. It is currently well documented that their use is associated with a high risk of central line-associated bloodstream infections (CLABSIs) in different patient populations and clinical settings, with increased rates of medical complications, hospitalizations, and healthcare costs. [1–3]

In attempts to prevent and reduce intravascular catheter-related infections, the U.S. Centers for Disease Control and Prevention (CDC) developed specific guidelines that are widely recognized as the document that better synthesizes current evidences on the delivery of care to patients with CVC, due to their acknowledged positive role in control and reduction of the infections related to the use of CVCs. [4, 5] Literature available so far shows that in different health contexts (and in different countries), health-care workers (HCW) are trained on the basis of the CDC recommendations, in which the major tools include frequent hand hygiene, barrier precautions, use of chlorhexidine for skin antisepsis, optimal catheter site selection, daily review of line necessity, and HCWs’ education and training. [6]

However, in spite of these recommendations, results of few epidemiological studies that have been conducted in various countries regarding the knowledge, attitudes, and practices among HCWs showed evidence of various deficiencies about the maintenance of CVCs, while several researches are highlighting the importance of a continuous monitoring of the implementation of the CDC recommendations themselves. [7–10] In Italy, to the best of our knowledge, there has been little information available addressing HCWs’ knowledge, attitudes, and practices regarding CVCs use. [11, 12]

In-depth knowledge of the issue about CVCs use by HCWs is of special relevance. Therefore, the aims of this study were to assess the level of knowledge, attitudes, and evidence-based practices about the maintenance of CVCs among a sample of HCWs’ working in different hospital wards in Italy and to highlight the main factors associated with these outcomes.

## Methods

### Enrollment

From July 2016 through January 2017, a cross-sectional survey was conducted in five public hospitals that adopted CDC guidelines (two General Hospitals, two Hospital Units, and an Institute of Research and Treatment), randomly selected from the list of all hospitals in the Campania Region, Italy. In order to obtain permission to carry out the survey in their institutions, hospital managers received a formal letter about description and objectives of the research. Subsequently, a random sample of HCWs employed in wards of surgery, medicine, nephrology, oncology, and emergency and intensive care units was selected. Each selected HCW received a letter of invitation reporting the study presentation, encouraging to participating in the survey, a copy of the questionnaire, the informed consent form, and an envelope to return the completed questionnaire. The HCWs were informed that their participation was voluntary, that all information gathered would be anonymous, and that confidentiality would be maintained by omitting any personal identifying information from the questionnaire. All participants provided written informed consent at the beginning of the survey prior to answering any question by reading the consent form. The sample size was determined before the study initiation. A formula for estimating a single population proportion with the assumption of a 95% confidence interval (CI), a margin of error of 5%, and an assumed prevalence of 70% of respondents having an accurate level of knowledge about the prevention of CVC-related infections, in accordance with the literature. [7, 12] Consequently, a sample of 323 HCWs was sought. The sample size was increases to 549 HCWs, to compensate a response rate of 70%, equally sampled amongst the eligible hospitals.

### Survey instrument

A structured self-administered questionnaire was designed to assess HCWs’ socio-demographic and professional characteristics, knowledge, attitudes, and behaviors about the insertion, maintenance of CVCs, and the prevention of CVC-related infections.

The questionnaire comprised a series of questions designed to assess the following items: (1) HCWs’ demographics and personal and professional characteristics (gender, age, highest educational qualification, professional role, number of years in practice, ward of activity, and number of years in the specific ward); (2) their knowledge of general measures regarding healthcare-associated infections and CLASBIs prevention, about the recommendations for the insertion of CVCs, about replacement regime of insertion site’s dressing, about timing of administration’s sets replacement, and about skin’s disinfection after insertion; (3) their attitudes towards perception of the utility of guidelines to prevent CLASBIs, perceptions about the patients’ infections risk, attitudes about the infections’ prevention with routinely substitution of CVC; (4) their behaviors and practices about the procedures of CVCs’ insertion and management, about patients’ clinical evaluation and of dressings’ status; (5) their sources of information and self-estimated educational needs. Questions used in the survey and response options are reported in (Additional file 1: Appendix 1).

### Content validity

The questionnaire was pre-tested and piloted with a convenience sample of 30 HCWs similar to the study population, who were interviewed to gauge feedback on the overall acceptability of the questionnaire in terms of length, clarity, and question formats. Based on respondents’ suggestions, some minor revisions included changes to the questionnaire items wording and format. Once data collection was completed, the researchers stored the data into a spreadsheet and data cleaning was performed to reduce the risk of error. Cronbach’s coefficient alpha (α) and average inter-item correlation (r) were measured to gauge the internal consistency of questions that loaded onto the same factors. Construct validity was explored in factor analysis, using Principal Components Analysis reported in (Additional file 2: Appendix 2).

### Ethics

The Ethical Committee of the Authors’ Institution approved the research protocol, the survey instrument, and the informed consent form.

### Statistical analysis

The statistical analysis was conducted in two steps following the strategy for model-building suggested by Hosmer et al. [13] Firstly, a univariate analysis was performed to assess the association of the independent variables with the outcomes of interest by using Student t test for independent samples, analysis of variance, and chi-square (*χ*^*2*^) test and the variables with a *p*-value equal or less than 0.25 were considered for possible introduction in the multivariate linear, logistic, and ordinal regressions models. Secondly, the following models were constructed: knowledge about CDC main recommendations for preventing CVC-related CLABSIs (educating and training healthcare personnel, the use maximal sterile barrier precautions, the use of chlorhexidine for antisepsis, the avoiding routine replacement of CVCs) (Model 1); perception of the utility of CDC guidelines (Model 2); adherence about the recommendations for CLASBIs prevention (Model 3); not removing catheter in presence of fever and performing dressing removal to allow examination of the site (Model 4); and behavior, in the presence of tenderness at catheter insertion site, about the examinations of the site (Model 5). Outcome variables originally consisting of multiple categories were dichotomized into two levels for the purpose of analysis.

In all models, the following independent variables were included: gender (male = 0, female = 1), age (continuous, in years), education level (secondary school or registered nurse diploma = 0, college degree or higher = 1), professional role (nurse = 0, physician = 1), number of years in practice (continuous, in years), hospital setting (General Hospital = 1, Hospital Unit = 2, Institute of Research and Treatment = 3), ward of activity (intensive care units = 1, other = 0), number of beds in the ward (continuous), previous attendance to educational courses about maintenance of CVCs (no = 0, yes = 1), availability of hospital internal protocols about CVCs management (no = 0, yes = 1), CDC guidelines as a source of information (no = 0, yes = 1), and need of additional information about the prevention of CVC-related infections (no = 0, yes = 1). The variables knowledge of the main CDC recommendations for preventing CVC-related infections (no = 0, yes = 1), knowledge of correct catheter site dressing regime replacement (no = 0, yes = 1), and knowledge of correct use of antibiotic ointments at the insertion site of CVC (no = 0, yes = 1) were included in models 2, 3, 4, and 5; knowledge of correct replacement timing of catheters inserted when adherence to aseptic technique is not ensured (no = 0, yes = 1), and HCWs’ perception of the utility of guidelines (low utility = 0, high utility = 1) were included in models 3, 4, and 5.

A stepwise backward selection procedure was used; a significance level of .20 was used as the criterion for variables to enter in the final multivariate regression models and *p*-value < .40 for exiting. In the logistic and ordered logistic regression models, adjusted odds ratios (ORs) and 95% CIs expressed the effect estimates. For the dependent variable measured on an ordinal scale, the approach followed the one proposed by Armstrong et al. [14] and the analysis was conducted choosing a cumulative odds model (ordered logistic regression models), for facilitating the interpretation in term of ORs. Standardized regression coefficients (*β*) were presented in linear regression model. All statistical tests were two-tailed and differences were considered to be statistically significant at a *p*-value equal or less than .05 throughout the whole study. Data were analyzed using Stata 14 statistical software [15].

## Results

Internal reliability estimates of the survey suggested a high degree of internal consistency for the investigated items (Cronbach’s α = 0.83 and *r* = 0.46). Of the 549 questionnaires distributed in the selected hospitals, a total of 378 HCWs agreed to participate in the survey, for a total response rate of 68.9%; 14 questionnaires were excluded from the analysis because of the large percentage of missing values (more than 40%) or when filled by training nurses. Socio-demographics and professional characteristics of the study population are reported in Table 1.

**Table 1:** Characteristics of respondent healthcare workers (HCW), *n* = 364

Characteristic	No.^a^ (%)	Mean ± SD°
Gender		
Male	180 (50.3)	
Female	178 (49.7)	
Age, in years		46.1 ± 9.7
Education level		
College degree or higher	201 (55.5)	
Other	161 (44.5)	
Professional role		
Nurse	254 (70.0)	
Physician	109 (30.0)	
Number of years in practice		19.8 ± 9.8
Hospital setting		
General Hospital	116 (31.9)	
Hospital Unit	141 (38.7)	
Institute of Research and Treatment	107 (29.4)	
Area of practice		
Medicine wards	139 (38.2)	
Surgery wards	115 (31.6)	
ICUs	110 (30.2)	
Previous attendance to specific training or courses about maintenance of CVCs	167 (46.7)	
Use of CDC guidelines as source of information	155 (42.6)	
Declared need of additional information about the prevention of CVC-related infections	290 (82.4)	

*ICUs*, intensive care units; *CVC*, central vascular catheter; *CDC*, U.S. Centers for Disease Control and Prevention

^a^Number for each item may not add up to total number of study population due to missing value

*°* Mean ± Standard deviation

The results regarding the knowledge of guidelines recommendations indicated that only 50.2% of HCWs knew that the routine replacement of CVCs do not prevent infections, whereas 45.1 and 35.5% correctly answered regarding the change of clean dressings on catheter insertion site for transparent dressings (every 7 days) and the for sterile gauzes (every 2 days). Two-thirds of respondents (63.8%) indicated 2% chlorhexidine preparation with alcohol as recommended for clean skin before CVC insertion and during dressing changes, while 16% indicate povidone-iodine base solutions. The vast majority of the sample acknowledged that before the insertion and maintenance of CVCs are needed hand hygiene procedures (93.4%) and the use of sterile gloves (83.5%). One-fifth (23.6%) indicated that an antibiotic ointment is recommended at the insertion site of CVC. More than half (53.9%) knew that, if the adherence to aseptic technique cannot be ensured (e.g., with catheters inserted during an emergency), the catheter should be replaced within 48 h. Overall, participants were poor knowledgeable regarding the replacement of administration sets since only 11.3 and 19.2% answered correctly that, respectively, infusion sets used for standard administrations should be changed every 96-h intervals, while tubing used to administer blood, blood products, or fat emulsions should be replaced within 24 h of initiating the infusion. The results of the multivariable logistic regression analysis showed that the knowledge about the CDC main recommendations (educating and training healthcare personnel, the use maximal sterile barrier precautions, the use of chlorhexidine for antisepsis, the avoiding routine replacement of CVCs) was higher in nurses (OR = 0.48), in those with a higher number of years of practice (OR = 1.04), who worked in hospitals with protocols about CVCs management (OR = 3.79), and who used guidelines as source of information (OR = 2.96) (Model 1 in Table 2).

**Table 2:** Multivariate regression models indicating associations between variables and outcomes of interest

Variable	OR	SE	95% CI	*P* value
Model 1: Knowledge about CDC main recommendations for preventing CVC-related CLABSIs				
Log likelihood = 133.20; *χ*^*2*^ = 54.62 (5 *df*); *p* < 0001				
Availability of internal protocols about CVC management	3.79	1.36	1.88–7.65	< .001
Use of CDC guidelines as source of information	2.96	0.99	1.54–5.69	.001
Number of years of practice	1.04	0.02	1.01–1.07	.03
Professional role (nurse)	0.48	0.18	0.23–0.99	.05
Gender (female)	1.68	0.54	0.89–3.17	.11
Variable	Coefficient	SE	t	P value
Model 2: Positive attitude of HCWs towards the utility of CDC guidelines				
*F* (6,293) = 7.23; *P* = < .0001; *R*^*2*^ = 0.13%; adjusted *R*^*2*^ = 0.11%				
Knowledge of correct use of antibiotic ointments at the insertion site of CVC	0.90	0.24	3.82	< .001
Knowledge about CDC main recommendations for preventing CVC-related infections	0.71	0.26	2.70	.007
Need of additional information about the prevention of CVC-related infections	0.62	0.27	2.29	.02
Professional role (physicians)	0.44	0.22	2.03	.04
Availability of internal protocols about CVC management	0.33	0.21	1.57	.12
Age	0.01	0.01	1.21	.23
Variable	OR	SE	95% CI	P value
Model 3: HCWs’ adherence about recommended evidence-based practices for preventing CLASBIs				
Log likelihood = − 164.05; *χ*^*2*^ = 10.02 (3 *df*); *p* = .02				
Professional role (physicians)	1.91	0.53	1.10–3.30	.02
Need of additional information about the prevention of CVC-related infections	2.10	0.84	0.96–4.61	.06
Availability of internal protocols about CVC management	1.44	0.39	0.85–2.44	.18
Variable	OR	SE	95% CI	P value
Model 4: HCWs with positive attitude towards not removing catheter if patient has fever and perform firstly the insertion site dressing removal to allow thorough examination of the site				
Log likelihood = − 154.77; *χ*^*2*^ = 21.61 (5 *df*); *p* = .0006				
Knowledge of correct use of antibiotic ointments at the insertion site of CVC	2.77	1.10	1.28–6.01	.01
Higher number of years of practice	1.03	0.02	1.00–1.06	.04
Knowledge of correct catheter site dressing regime replacement	1.92	0.62	1.01–3.63	.05
Higher educational level (college degree or higher)	1.79	0.52	1.01–3.18	.05
Availability of internal protocols about CVCs management	1.48	0.02	0.85–2.57	.17
Variable	OR	SE	95% CI	P value
Model 5: Correct behaviour regarding the removal of catheter dressing if patients have tenderness at palpation and the performing of a thorough examination of the site				
Log likelihood = − 294.28; *χ*^*2*^ = 25.27 (4 *df*); *p* < .0001				
Age	1.03	0.01	1.01–1.06	.02
Knowledge about CDC main recommendations for preventing CVC-related infections	2.23	0.77	1.13–4.40	.02
Perception of high utility of guidelines	1.79	0.51	1.03–3.12	.04
Professional role (physicians)	1.72	0.49	0.98–3.02	.06

*HCWs*, healthcare workers; *CVC*, central vascular catheter; *CDC*, U.S. Centers for Disease Control and Prevention; *CI*, confidence interval; *OR*, odds ratio; *SE*, standard error; *df*, degrees of freedom

The respondents’ attitudes towards the utility of guidelines for preventing intravascular catheter-related infections procedures, measured on a ten-point Likert scale ranging from 1 to 10, with higher scores indicating more positive attitude, showed a mean score for the whole sample of 8.7 (SD = 1.8). The results of the fully adjusted multivariable linear regression model indicated that a higher attitude was been observed in physicians, in those who knew that antibiotic ointment at insertion site of CVC does not decrease the risk for catheter-related infections and causes antibiotic resistance, in HCWs that acknowledge CDC main recommendations for preventing CLABSIs, and in those needing additional information about the prevention of CVC-related infections (Model 2 in Table 2). Finally, 78.4% agreed that the CVC could cause serious infective complications, 62.0% reported the useful of palpation to verify infection signs and one-third believe not correctly useful to remove CVC with only fever.

Regarding the behaviors, almost all HCWs (93.4%) self-reported that they performed hand hygiene in their working activity. Less than three forth (72.7%) declared a constant visual monitoring of the catheter site when changing the dressing, only 45.8% of them performed the palpation through an intact dressing on a regular basis, and 66.7% remove the site dressing if patients have tenderness. Regarding the presence of fever without obvious source, about a half of HCWs reported that they remove the site dressing to examine the site itself. Finally, 60.4% encouraged patients to report any changes in their catheter site or any other discomfort manifestations suggesting local or bloodstream infection.

The multiple logistic regression analysis showed that the investigated evidence-based practices to prevent the CLABSIs were more likely to be performed by physicians (OR = 1.91) (Model 3 in Table 2).

Instead, the strongest predictors of attitude towards not removing catheter if patient has fever and perform firstly the insertion site dressing removal to allow thorough examination of the site, suggested by the specific multivariable logistic regression analysis, were the higher number of years of practice (OR = 1.03), a higher level of education (OR = 1.79), the knowledge about catheter site dressing regime replacement (OR = 1.92), and of the correct antibiotic ointment use at the insertion site of CVCs (OR = 2.77) (Model 4 in Table 2).

The final model of the multivariate ordinal logistic regression analysis examining the variables associated about the correct behavior regarding the removal of catheter dressing if patients have tenderness at palpation and the performing of a thorough examination of the site, measured through a 5-point Likert-type scale ranging from “never” to “always”, showed that this practice was more likely in older HCWs (OR = 1.03), in whom had a correct knowledge of CDC main recommendations for preventing CVC-related CLABSIs (OR = 2.23), and in those who perceived a higher utility of guidelines (OR = 1.79) (Model 5 in Table 2).

Among the entire sample, only 104 HCWs (28.6%) performed catheter-insertion: practices of this subgroup are listed in Table 3.

**Table 3:** Health-care workers’ practices in central vascular catheters (CVC) insertion, *n = 104*

Professional Role	*n* ^a^ *(%)*
Physicians	52 (50.0%)
Nurses	52 (50.0%)
Practices	
Perform hand hygiene	97 (96.0%)
Wear sterile gloves	79 (76.7%)
Use new sterile gloves when changing the CVC	48 (47.1%)
Wear sterile gown	51 (50.0%)
Use of mask	60 (58.3%)
Use of body drape	68 (66.7%)
Operate in dedicated room	49 (48.5%)
Not apply antibiotic ointment	33 (32.4%)

^a^Number for each item may not add up to total number of study population due to missing value

## Discussion

In this cross-sectional survey important results were yielded regarding knowledge, attitudes, and practices about the CLABSIs prevention amongst a random sample of HCWs in Italian hospitals.

The self reported responses indicated the current state of knowledge related to CDC main recommendations was low, being the 20.7% of HCWs knowledgeable about them. Analysis of the predictors of being more knowledgeable showed that there was a significant difference in the level of knowledge according to the presence of hospital internal protocols, the use of guidelines as source of information, and working from a higher number of years. These associations may be explained clearly by the higher experience level in HCWs working from a higher number of years, as well as by the fact that the presence, in the place of work, of written protocols and pathways increases the HCWs’ compliance to the prescribed recommendations. These findings suggest the need of educational programs in order to improve the level of knowledge. [16]

HCWs had an extremely positive attitude towards the utility of the CDC guidelines for preventing CLABSIs and this is in accordance with a previous study conducted in Italy regarding the attitudes toward guidelines. [17] This attitude was more likely observed in physicians, in those who knew CDC main recommendations and the correct use of antibiotic ointments, and who declared the needing for additional information about CLABSIs prevention procedures. Furthermore, contrarily to the findings of Bianco et al. [12], the attitude was shown as independent of HCWs’ ward of activity, even if guidelines emphasize the importance of the prescribed indications mostly in ICUs, because of the highest risk of CLABSIs in their patients. [18]

The analysis of the surveys revealed a varied distribution of practices performed by the interviewed HCWs. The most noticeable data is the lack of implementation of the evidence-based recommendations for the surveillance of the catheter insertion site: [19] only physicians were more likely to perform these practices.

More particularly, different results were shown considering the practices implemented by HCWs when patients experience tenderness at the insertion site at palpation through the intact dressing or fever without obvious source: in these cases the main suggested procedure is to initially remove the catheter dressing to allow thorough examination of the site. [20, 21] A key objective of this research was to investigate the independent predictive factors for correct practices adoption in these mentioned cases. HCWs with more years of practice and other evidence-based knowledges on CLABSIs prevention, such as the avoiding of antibiotic ointment use and the dressing replcemente regimes, are more likely to correctly manage patients with fever. The same predictors were found in HCWs with university curricula: while the higher quality of education ensure the achieving of more information on topic of HAIs and its prevention, confirming that healthcare professionals, if experienced, have an average knowledge level higher on these topics [9, 10]. More interesting is the finding that this behaviour changed significantly with the knowledge of other specific CDC recommendations, even when the responses did not suggest a clear association with the use of guidelines as source of information. Regarding HCWs’ practices in case of tenderness at CVC site, instead, the appropriate procedures of not removing catheter and performing dressing removal are more likely if executed by aged HWCs, and, more interesting, if executed by personnel with the correct knowledge of CDC main recommendations on CLABSIs control, as well as in whom percieved the higher utility of guidelines themselves.

In the all sample, 28.6% of the interviewed reported to insert CVCs: a scarce level in the application of evidence-based recommendations, as listed in Table 3, was shown in this subgroup, in concordance with the results reported by Alkubati et al., [22] but displayed a lower level of proper practices if compared with a recently published research conducted in Campania area just few months after the publication of CDC guidelines. [11]

It is also worth pointing out the role of HCWs-oriented educational intervention programs for reducing CLABSIs: independent analysis showed significant associations between the attendance of specific courses and several items of the survey, such as the knowledge of avoiding routine replacement of central venous catheters as a strategy to prevent infection, knowledge of 2% chlorhexidine skin preparation with alcohol as indicated antiseptic, knowledge of correct dressing replacement regimes, the perception of high utility of guidelines and their use as source of information. Mentioning the implementation of evidence-based practices, the participating in trainings or continuing educational courses is significantly associated only with the attitude of not removing CVC if patient had fever and examine the insertion site; whereas no significant association was found with the outcomes in model 3 and 5. These finding confirmed the important role played by education and formal training of healthcare staff regarding the indication for maintenance of the CVCs. [23, 24]

The study reflected, similarly to other research, [7, 9, 25] a consistent lack of CDC guidelines implementation, while non-evidence-based knowledge and behaviours are still widespread in clinical practice and a full adapting of guidelines for preventing CLABSIs to local context is further to be achieved. In addition, as a positive aspect, the resulting data validated the information that a higher education level, years of experience, formal training and specific courses, and the presence of hospital protocols favorably modify HCWs’ knowledge, attitude and practices towards the topics of this research.

Guideline implementation is complex and the translation of recommendations to clinical practice involves many steps and participants. [26] Nevertheless, the results of the presented study made it possible to draw firm suggestions for the future improvements in CLABSIs control. Findings showed that the sub-optimal HCWs’ level of knowledge represents one of the major weaknesses of CLABSIs prevention and control; the planning of future interventions on this topic should incorporate adequate training schemes for improving HCWs’ preparedness and professional development, also through specific training courses and continuing medical education programs. Another possible task to be planned, as a responsibility for health facilities, is about structuring integrated care pathways in order to clearly translate CDC guidelines into the detailed steps that are necessary in the care of patients with CVC [27]. Indeed, as the findings from this research showed, the availability of internal protocols about CVC management is a predictor for better knowledge of the CDC recommendations. At the same time, particularly in the Italian framework, health service providers’ efforts are needed in enhance the importance of following evidence based recommendations, in the light of the last Medical Liability Reform of 2017, which strengthened the guidelines role itself in clinical practice [28].

To precisely appreciate the findings of this study, some potential limitations must be considered. First, the cross-sectional nature carries with it the disadvantage of not being able to prospectively determinate a causal effect of detected items. Second, potential bias due to the use of a self-report survey is necessary, where respondents could be influenced by thinking at researchers’ expectations or hide the actual incorrect behaviors; the anonymous and not trackable questionnaire likely softened this limitation. Third, whilst the data found an inadequate adherence to the guidelines’ evidences, it’s not possible assess the barriers to this mis-implementation, also for a likely multi-factorial nature [29, 30]: such an issue may foster further researches. Despite these limitations, this survey provides important findings that can be considered generalizable because the sample had been carefully selected, the methodology of the study is accurate, and the response rate was satisfactory.

## Conclusions

In conclusion, the presented research highlights a lack of evidence-based knowledge and practices regarding the management of CVCs, despite the self-reported positive attitude towards the specific guidelines. This survey also indicates the baseline role of education and training programs to improve knowledge, as well as the role of organizational interventions to address the adherence to best procedures suggested by evidences for the reduction of CLABSIs and for patient safety.

## Additional files

Additional file 1: **Appendix 1.*** Survey****.*** Questions used in the survey. (DOC 55 kb)

Additional file 2: **Appendix 2.*** Results of Principal Components Analysis and Factor Analysis****.*** Principal Components Analysis and Scree-Plot of the Eigenvalues performed to explore construct validity of the survey. (DOC 44 kb)

## Abbreviations

CDC: Centers for Disease Control and Prevention
CLABSI: Central line-associated bloodstream infections
CVC: Central venous catheters
HCWs: Healthcare workers
