# Supplementary material for: The adherence to guidelines for preventing CVC-related infections: a survey among Italian health-care workers
Source: BMC Infect Dis. 2018 Dec 3;18:606. doi: 10.1186/s12879-018-3514-x (PMC6276178; doi:10.1186/s12879-018-3514-x)
Supplement: Supplementary file 1 — Appendix 1. Survey. Questions used in the survey. (DOC 55 kb) [file 12879_2018_3514_MOESM1_ESM.doc]

**The adherence to guidelines for preventing CVC-related infections:**

**a survey among Italian health-care workers**

**Appendix 1 – Survey**

*Questions used in the survey*

**A. DEMOGRAPHIC AND PRACTICE CHARACTERISTICS OF THE RSPONDENT HCWs**

A1. Gender: □ Male □ Female

A2. Year of birth _____

A3. What is your highest educational level? □ Secondary school □ Registered nurse diploma □ College degree □ Higher (specify_____)

A4. Which is your professional role? □ Nurse □ Physician

A5. How many years have you been working? _____

A6. In what kind of hospital do you work? □ General Hospital □ Hospital Unit □ Institute of Research and Treatment

A7. In what kind of ward do you work in the hospital? □ Intensive care unit □ Medicine ward □ Surgery ward

A8. How many years have you been working in this ward? _____

A9. How many beds are the in the ward where you are working? _____

**B. Knowledge**

B1. Have you ever attend educational courses about maintenance of CVCs? □ Yes □ No

B2. Please, indicate an option for each of the following items:

| - Insertion and maintenance of CVCs must be preceded hand hygiene procedures | □ Yes □ No □ Don’t know |
| --- | --- |
| - Insertion and maintenance of CVCs must be performed using sterile gloves | □ Yes □ No □ Don’t know |
| - The use of antibiotic ointment at CVC insertion site is recommended for reducing infections | □ Yes □ No □ Don’t know |
| - If the adherence to aseptic technique cannot be ensured during the CVC insertion (e.g., with catheters inserted during an emergency), the catheter should be replaced within 48 hours | □ Yes □ No □ Don’t know |
| - The routine replacement of central venous catheters is a recommended strategy to prevent infection? | □ Yes □ No □ Don’t know |

B3.How often should it be replaced a clean and intact transparent dressings on catheter insertion site? □ every 24h □ every 2 days □ every 7 days □ every 4 days □ don’t know

B4. How often should it be replaced a clean and intact sterile gauzes on catheter insertion site? □ every 24h □ every 2 days □ every 7 days □ every 4 days □ don’t know

B5. How often should they be replaced administration sets used for standard infusions? □ every 12h □ every 24h □ every 48h □ every 72h □ every 96h □ don’t know

B7. How often should they be replaced administration sets used to administer blood, blood products, or fat emulsions? □ every 12h □ every 24h □ every 48h □ every 72h □ every 96h □ don’t know

B8.What is recommended for clean skin before CVC insertion and during dressing changes? □ 2% chlorhexidine preparation with alcohol □ povidone-iodine base solution □ quaternary ammonium preparation □ other______ □ don’t know

**C. Attitudes**

C1. How would you rate the utility of CDC *Guidelines for the prevention of intravascular catheter-related infections* on a 1 to 10 scale with 1 meaning “no useful at all” and 10 “very useful”?

(Not useful at al) 1 2 3 4 5 6 7 8 9 10 (Very useful)

C2. Please, indicate your opinion about the following items:

| - CVC is a device that could cause serious infective complications | □ Agree □ Not agree □ Don’t know |
| --- | --- |
| - Palpation at catheter insertion site is useful to verify infection signs | □ Agree □ Not agree □ Don’t know |
| - It’s useful promptly removing CVC when patients experience fever | □ Agree □ Not agree □ Don’t know |

**D. behaviours and practices**

D1. Do you perform CVCs insertion? □ Yes □ No

*If you do not, please go to question D3.*

D2. When you are performing CVCs insertion, how often do you…?

| - wash your hands before the insertion? | □ Always □ Very Frequently  □ Occasionally □ Rarely □ Never |
| --- | --- |
| - wear sterile gloves? | □ Always □ Very Frequently  □ Occasionally □ Rarely □ Never |
| - use new sterile gloves when changing the CVC? | □ Always □ Very Frequently  □ Occasionally □ Rarely □ Never |
| - wear sterile gown? | □ Always □ Very Frequently  □ Occasionally □ Rarely □ Never |
| - use mask? | □ Always □ Very Frequently  □ Occasionally □ Rarely □ Never |
| - use a body drape for the patient? | □ Always □ Very Frequently  □ Occasionally □ Rarely □ Never |
| - operate in dedicated room? | □ Always □ Very Frequently  □ Occasionally □ Rarely □ Never |
| - apply antibiotic ointment? | □ Always □ Very Frequently  □ Occasionally □ Rarely □ Never |

D3. Before replacing catheter site dressing, do you perform hand hygiene? □ Yes □ No

D4. In patient caring, how often do you…?

| - visual monitor the catheter site when changing the dressing? | □ Always □ Very Frequently  □ Occasionally □ Rarely □ Never |
| --- | --- |
| - performed the palpation of the site through the intact dressing on a regular basis? | □ Always □ Very Frequently  □ Occasionally □ Rarely □ Never |
| - remove the site dressing if patients have tenderness to allow thorough examination of the site? | □ Always □ Very Frequently  □ Occasionally □ Rarely □ Never |

D5. If patient has fever, what do you do?

□ Remove the site dressing to examine the site □ Remove the site dressing to allow thorough examination of the site □ Run a blood culture □ Other (specify____)

**E. Source of information**

E1. Are there in your hospital internal care protocol on the prevention of intravascular catheter-related infections? □ Yes □ No

E2. Do you utilize CDC Guidelines for the prevention of intravascular catheter-related infections as a source of information? □ Yes □ No

E3. Do you feel you need additional information about the prevention of CVC-related infections? □ Yes □ No
