# Supplementary material for: The adherence to guidelines for preventing CVC-related infections: a survey among Italian health-care workers
Source: BMC Infect Dis. 2018 Dec 3;18:606. doi: 10.1186/s12879-018-3514-x (PMC6276178; doi:10.1186/s12879-018-3514-x)
Supplement: Supplementary file 2 — Appendix 2. Results of Principal Components Analysis and Factor Analysis. Principal Components Analysis and Scree-Plot of the Eigenvalues performed to explore construct validity of the survey. (DOC 44 kb) [file 12879_2018_3514_MOESM2_ESM.doc]

**The adherence to guidelines for preventing CVC-related infections:**

**a survey among Italian health-care workers**

**Appendix 2**

*Results of Principal Components Analysis and Factor Analysis*

In order to check content validity of the survey instrument designed for the purposes of the study, Principal Components Analysis (PCA) and Factor Analysis (FA) were conducted on variables that point back to the outcomes of interest used in the multivariate regression models:

1. *Knowledge about CDC main recommendations for preventing CVC-related CLABSIs*
2. *Positive attitude of HCWs towards the utility of CDC guidelines*
3. *HCWs’ adherence about recommended evidence-based practices for preventing CLASBIs*
4. *HCWs with positive attitude towards not removing catheter if patient has fever and perform firstly the insertion site dressing removal to allow thorough examination of the site*
5. *Correct behaviour regarding the removal of catheter dressing if patients have tenderness at palpation and the performing of a thorough examination of the site*.

Results are presented in Table S1 and Figure S1.

| Table S1. **Results of Principal Components Analysis and Factor Analysis** | | | | |
| --- | --- | --- | --- | --- |
| ***Eigenvalues of the principal components*** | | | | |
|  | Eigenvalue | Difference | Proportion of variance explained | Cumulative variance explained |
| 1 | 1.57 | 0.43 | 0.31 | 0.31 |
| 2 | 1.14 | 0.21 | 0.23 | 0.54 |
| 3 | 0.93 | 0.11 | 0.19 | 0.73 |
| 4 | 0.82 | 0.29 | 0.16 | 0.89 |
| 5 | 0.53 | - | 0.11 | 1.00 |
| ***Factor loadings*** | | | |  |
| Variables |  | Factor1 | Factor2 |  |
| 1 |  | 0.34 | 0.72 |  |
| 2 |  | 0.36 | 0.61 |  |
| 3 |  | 0.70 | -0.46 |  |
| 4 |  | 0.81 | -0.21 |  |
| 5 |  | 0.45 | 0.06 |  |
| *Eigenvalue* represents the variance of the component and is considerate to be critical for values > 1. All the values add to the sum of the number of the variables. The *Difference* is the size of a component’s eigenvalue and the next component’s eigenvalue. Thus, the first two principal components with eigenvalues > 1 explained the 54% of the information in the data set.  Factor loadings reflected the multiplicity of the survey. | | | | |

Figure S1. **Scree Plot of the Eigenvalues**

*PCA*, Principal Components Analysis; *Eigenvalue*, variance of the component;

*95% CI*, Confidence interval
